# Characterisation of phospholipid: diacylglycerol acyltransferases (PDATs) from *Camelina sativa* and their roles in stress responses

**DOI:** 10.1242/bio.026534

**Published:** 2017-07-05

**Authors:** Lixia Yuan, Xue Mao, Kui Zhao, Xiajie Ji, Chunli Ji, Jinai Xue, Runzhi Li

**Affiliations:** 1 Institute of Molecular Agriculture and Bioenergy, Shanxi Agricultural University, Taigu 030801, Shanxi, China; 2 College of Biological Science and Technology, Jinzhong University, Jinzhong 030600, Shanxi, China

**Keywords:** *Camelina sativa*, Phospholipid diacylglycerol acyltransferase (PDAT), Oil and fatty acid biosynthesis, Transient expression, Stress response

## Abstract

As an important oilseed worldwide, *Camelina sativa* is being increasingly explored for its use in production of food, feed, biofuel and industrial chemicals. However, detailed mechanisms of camelina oil biosynthesis and accumulation, particularly in vegetative tissues, are understood to a very small extent. Here, we present genome-wide identification, cloning and functional analysis of phospholipid diacylglycerol acyltransferase (PDAT) in *C. sativa*, which catalyses the final acylation step in triacylglycerol (TAG) biosynthesis by transferring a fatty acyl moiety from a phospholipid to diacylglycerol (DAG). We identified five genes (namely *CsPDAT1-A*, *B*, and *C* and *CsPDAT2-A* and *B*) encoding PDATs from the camelina genome. *CsPDAT1-A* is mainly expressed in seeds, whereas *CsPDAT1-C* preferentially accumulates in flower and leaf tissues. High expression of *CsPDAT2-A* and *CsPDAT2-B* was detected in stem and root tissues, respectively. Cold stress induced upregulation of *CsPDAT1-A* and *CsPDAT1-C* expression by 3.5- and 2.5-fold, respectively, compared to the control. Salt stress led to an increase in *CsPDAT2-B* transcripts by 5.1-fold. Drought treatment resulted in an enhancement of *CsPDAT2-A* mRNAs by twofold and a reduction of *CsPDAT2-B* expression. Osmotic stress upregulated the expression of *CsPDAT1-C* by 3.3-fold. Furthermore, the cDNA clones of these *CsPDAT* genes were isolated for transient expression in tobacco leaves. All five genes showed PDAT enzymatic activity and substantially increased TAG accumulation in the leaves, with CsPDAT1-A showing a higher preference for ɑ-linolenic acid (18:3 ω-3). Overall, this study demonstrated that different members of CsPDAT family contribute to TAG synthesis in different tissues. More importantly, they are involved in different types of stress responses in camelina seedlings, providing new evidence of their roles in oil biosynthesis and regulation in camelina vegetative tissue. The identified CsPDATs may have practical applications in increasing oil accumulation and enhancing stress tolerance in other plants as well.

## Abstract (precis)

**Summary:** Five CsPDAT family members were identified from *Camelina sativa* and they contribute to TAG synthesis in different tissues and various stress responses, offering new targets for lipid metabolic engineering.

## INTRODUCTION

Camelina [*Camelina sativa* (L.) Crtz.] is an important Brassicaceae oilseed crop with several excellent qualities that are contributing to the rising interest in its use for food, feedstock, pharmaceuticals, biofuel and other industries (Betancor et al., 2015; Haslam et al., 2016). Camelina seeds accumulate high levels of oil (40%) and protein (30%), but less glucosinolates (toxic for humans and animals) than do other Brassicaceae (Vollmann and Eynck, 2015). Particularly, unsaturated fatty acids make up 90% of camelina oil, which includes 40% of ω-3 fatty acid (18:3), 25% of linoleic acid (18:2), 15% of oleic acid (18:1), and 15% of eicosenoic acid (20:1) (Yuan et al., 2015). This desirable fatty acid composition enables camelina to be developed as a nutritionally enhanced oil.

The superior characteristics of camelina include high resistance to diseases and pests, a short lifetime of about 80-100 days, low-input requirements, and strong adaptability to adverse environmental conditions (Berti et al., 2011; Zanetti et al., 2013; Zubr, 1997). Moreover, camelina can be easily transformed by *Agrobacterium*-mediated floral vacuum infiltration (Lu and Kang, 2008), permitting quick engineering for improved seed quality and agronomic traits at a higher efficiency than with other oilseeds. Together, these distinct features make camelina an ideal crop for sustainable and environmentally friendly production of low-cost vegetable oil. As the market for camelina is increasing, considerable efforts are needed to enhance camelina agronomics and genetic traits such as seed quality and overall plant growth and development. Understanding the molecular mechanisms responsible for oil biosynthesis and other biological processes would lay the foundation for camelina breeding and agronomic production.

Triacylglycerols (TAGs), glycerol esters of fatty acids, are the predominant components of plant oils, serving as the energy-storage lipids to be used during plant seed germination and seedling establishment, and also functioning as a major source of highly reduced carbon molecules for food, feed and fuel (Murphy et al., 2005). TAGs in plants can be synthesised via multiple processes involving a series of enzymes (Wu et al., 2012; Yuan et al., 2015). In the acyl-CoA-dependent Kennedy pathway, glycerol-3-phosphate (G3P) is sequentially acylated to form lyso-phosphatidic acid (LPA) via glycerol-3-phosphate acyltransferase (GPAT; EC 2.3.1.15) activity, which is then converted to phosphatidic acid (PA) via lyso-phosphatidic acid acyltransferase (LPAAT; EC 2.3.1.51) activity. Afterwards, PA is dephosphorylated by phosphatidic acid phosphatase (PAP; EC 3.1.3.4) to generate *sn-1,2*-diacylglycerol (DAG), followed by the final acylation at the *sn-3* position of *sn-1, 2*-DAG to produce TAG, catalysed by diacylglycerol acyltransferase (DGAT; EC 3.2.1.20) using acyl-CoA as the acyl donor (Bates et al., 2013; Chapman and Ohlrogge, 2012; Ohlrogge and Browse, 1995). The acyl-CoA-independent pathway of TAG synthesis is characterised by phospholipid:diacylglycerol acyltransferase (PDAT; EC 2.3.1.158) transferring the fatty acyl moiety from the *sn-2* position of a phosphatidylcholine (PC) to the *sn-3* position of *sn-1, 2*-DAG to form TAG (Dahlqvist et al., 2000; Liu et al., 2012; Ståhl et al., 2004). In addition, a DAG/DAG transacylase uses two molecules of DAG functioning as both acyl donor and acceptor to form TAG and monoacylglycerol (Stobart et al., 1997).

The final acylation catalysed by either DGAT or PDAT has been considered to be the rate-limiting step in TAG synthesis (Li et al., 2010, 2013). At least three distinct classes of DGATs, namely, DGAT1, DGAT2 (no sequence homology to DGAT1) and DGAT3(soluble enzyme) have been characterised in plants (Hernández et al., 2012; Routaboul et al., 1999; Saha et al., 2006; Shockey et al., 2006; Zou et al., 1999). The specific functions of these DGATs in TAG biosynthesis differ in different organisms and even in different tissues within the same species (Li et al., 2012; Shockey et al., 2006) despite data indicating the role of DGAT1 as a major player in mediating TAG biosynthesis in developing seeds (Routaboul et al., 1999; Zou et al., 1999) and senescent leaves (Slocombe et al., 2009) of Arabidopsis. Moreover, DGAT1 is elevated in both seeds and other vegetative tissues under stress. *DGAT1* transcripts were greatly increased in Arabidopsis leaves under stress-induced senescence (Kaup et al., 2002), and in Arabidopsis seedlings under conditions of low-nitrogen, treatment with abscisic acid (ABA) and other environmental stresses (Kong et al., 2013; Lu et al., 2003; Yang et al., 2011).

Compared with DGATs, relatively little functional information is available on PDATs (Pan et al., 2015), although PDATs have been identified from yeast (*Saccharomyces cerevisiae*; encoded by *LOR1* gene; Dahlqvist et al., 2000), Arabidopsis (AtPDAT1 and AtPDAT2; Ståhl et al., 2004), flax (*Linum usitatissimum*) (LuPDAT1 and LuPDAT2; Pan et al., 2013), castor bean *(Ricinus communis)* (RcPDAT1 and RcPDAT2; Kim et al., 2011), two green microalga *Chlamydomonas reinhardtii* (CrPDAT; Yoon et al., 2012) and *Myrmecia incise* (*MiPDAT*; Liu et al., 2016). In Arabidopsis, PDAT1 was not a key contributor of TAG content in developing seeds (Mhaske et al., 2005), but exhibited overlapping function with DGAT1 in TAG biosynthesis in seed and pollen grain development (Zhang et al., 2009). Previous studies indicate that PDAT can exist in multiple copies in plant genomes, and different PDATs may have different TAG synthesising abilities, highlighting the need for a deeper understanding of the complexity of plant PDATs. Particularly, the functional role of PDAT in non-seed tissues and in plant response to various stresses remains to be elucidated.

In this study, we have used bioinformatics tools to characterise the PDAT protein family in the genome of *C. sativa*, using its reference genome that became publicly available in 2014 (Kagale et al., 2014). Quantitative PCR was employed to detect expression profiles of *CsPDAT* members in various camelina tissues and also in response to various abiotic stresses. Furthermore, the cDNA clones of *CsPDAT* genes were isolated for transient expression in tobacco leaves, in order to investigate individual CsPDAT functions. Our present data demonstrate that different members of CsPDAT family function differently in TAG accumulation and in plant response to drought, cold, osmotic and salt stresses, thus providing further insight into the diversity of plant PDAT functions and the complicated regulatory mechanism of oil biosynthesis and accumulation in both seeds and vegetative tissues.

## RESULTS

### Characterisation of five members of the PDAT family from camelina genome

The completed camelina genome sequence database (Kagale et al., 2014) provided the starting point for identifying camelina genes homologous to known genes (such as *DGATs* and *PDATs*) involved in the final step of TAG synthesis. To identify the gene encoding PDAT in *C. sativa*, we conducted a BLAST search of the camelina genome database (www.ncbi.nlm.nih.gov/genome/?term=camelina+sativa) using amino acid (AA) sequences of Arabidopsis PDAT1 (At5g13640) and PDAT2 (At3g44830) as the query sequence. Consequently, five candidate camelina *CsPDAT* genes, namely *CsPDAT1-A*, *CsPDAT1-B*, *CsPDAT1-C*, *CsPDAT2-A* and *CsPDAT2-B*, with a low *P*-value (<2.3E-204) were identified from the database. The three CsPDAT1s, i.e. CsPDAT1-A, CsPDAT1-B and CsPDAT1-C, show 97%, 96% and 95% AA identity with AtPDAT1, respectively, while the two CsPDAT2s, i.e. CsPDAT2-A and CsPDAT2-B, show 90% and 89% AA identity with AtPDAT2, respectively.

**Fig. 1.**
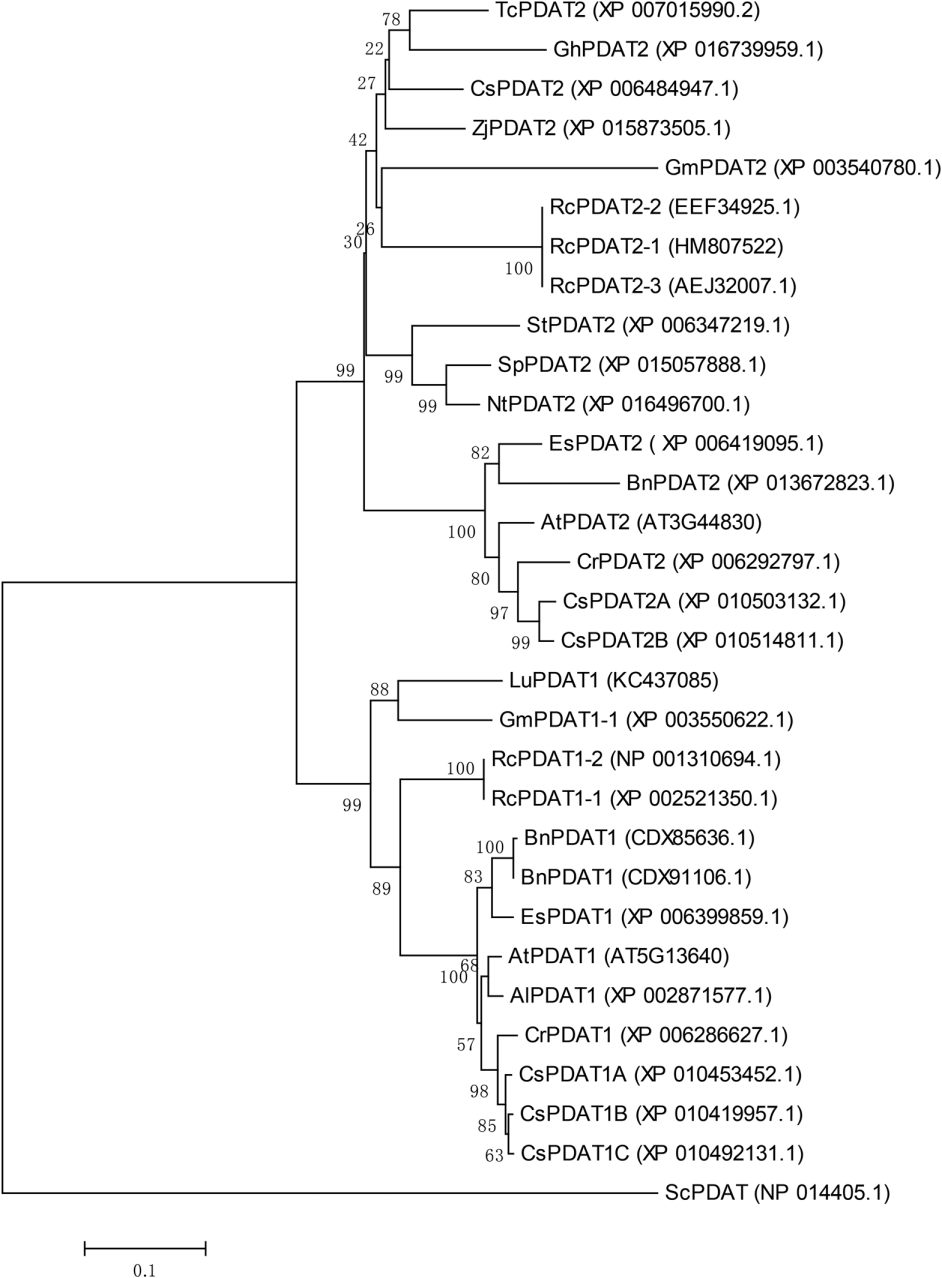
**Phylogenetic relationship of five CsPDATs and a number of known PDATs from other plants.** The ML tree was generated using the online program RAxML (http://embnet.vital-it.ch/raxml-bb/) under the contemporary model (JTT) of sequence evolution. Numbers above branches represent the support values (Bayesian posterior probabilities). The tree is rooted using PDAT sequences from *S. cerevisiae* as the outgroup. The scale bar represents the number of amino acid replacements per site. The protein sequences used here were AlPDAT1 (XP_002871577.1) from *Arabidopsis lyrata*; AtPDAT1 (AT5G13640; accession number: NP_196868.1) and AtPDAT2 (AT3G44830; accession number: Q9FYC7.1) from *Arabidopsis thaliana*; BnPDAT1 (accession number: CDX85636.1), BnPDAT1 (accession number: CDX91106.1), and BnPDAT2 (accession number: XP_013672823.1) from *Brassica napus*; CrPDAT1 (accession number: XP_006286627.1) and CrPDAT2 (accession number: XP_006292797.1) from *Capsella rubella*; CsPDAT1A (accession number: XP_010453452.1), CsPDAT1B (accession number: XP_010419957.1), CsPDAT1C (accession number: XP_010492131.1), CsPDAT2A (accession number: XP_010503132.1) and CsPDAT2B (accession number: XP_010514811.1) from *Camelina sativa*; CsPDAT2 (accession number: XP_006484947.1) from *Citrus sinensis*; EsPDAT1 (accession number: XP_006399859.1) and EsPDAT2 (accession number: XP_006419095.1) from *Eutrema salsugineum*; GhPDAT2 (accession number: XP_016739959.1) from *Gossypium hirsutum*; GmPDAT1-1 (accession number: XP_003550622.1) and GmPDAT2 (accession number: XP_003540780.1) from *Glycine max*; LuPDAT1 (accession number: KC437085) from *Linum usitatissimum*; NtPDAT2 (accession number: XP_016496700.1) from *Nicotiana tabacum*; RcPDAT1-2 (accession number: NP_001310694.1), RcPDAT1-1 (accession number: XP_002521350.1), RcPDAT2-3 (accession number: AEJ32007.1), RcPDAT2-2 (accession number: EEF34925.1) and RcPDAT2-1 (accession number: HM807522) from *Ricinus communis*; ScPDAT (accession number: NP_014405.1) from *Saccharomyces cerevisiae*; SpPDAT2 (accession number: XP_015057888.1) from *Solanum pennellii*; StPDAT2 (accession number: XP_006347219.1) from *Solanum tuberosum*; TcPDAT2 (accession number: XP_007015990.2) from *Theobroma cacao*; ZjPDAT2 (accession number: XP_015873505.1) from *Ziziphus jujube*.

General information about the identified *CsPDAT* genes is listed in Table 1. Gene structure examination showed that all the *CsPDATs* have six exons, except for *CsPDAT1-C* which has seven exons. All the introns had splicing consensus GT-AG junctions. In order to confirm that the sequences obtained using BLAST were certainly *PDAT* genes, phylogenetic analysis was performed using these candidate CsPDATs and a number of known PDATs from other plants (Fig. 1) (for the multiple sequence alignment of PDATS see the Supplementary Materials). All the PDATs tested could be distinctly classified into two groups, i.e. PDAT1 or PDAT2, based on their polypeptide sequences. The three CsPDAT1s formed a branch closely related to *Capsella rubella* PDAT1 (CrPDAT1) and Arabidopsis PDAT1 (AtPDAT1), whereas the two CsPDAT2s were clustered as a branch closely related to CrPDAT2 and AtPDAT2.

**Table 1.**
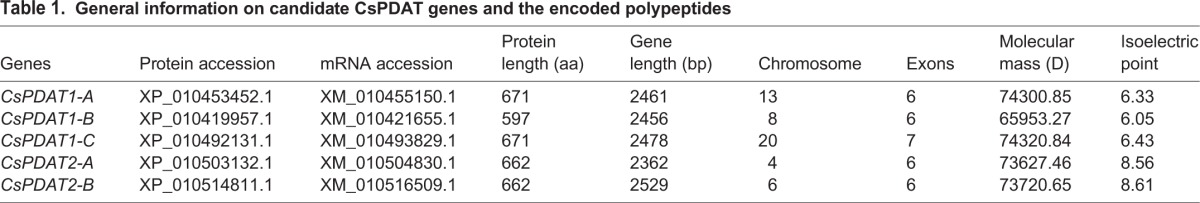
**General information on candidate CsPDAT genes and the encoded polypeptides**

TMHMM analysis (transmembrane prediction based on a hidden Markov model) and hydropathy profiling indicated that CsPDATs are integral membrane proteins with a transmembrane domain (TMD) at their N-termini, similar to the structures of yeast and Arabidopsis PDATs (Ghosal et al., 2007; Yoon et al., 2012), thus showing a highly conserved TMD position in the PDATs (Pan et al., 2015). In addition, an aromatic amino acid-rich stretch was identified at the C-terminal end of the CsPDATs, which may act as an ER localisation signal that is found in other known acyltransferases as well (McCartney et al., 2004; Liu et al., 2012).

Further characterisation of CsPDATs using InterPro and Pfam analysis revealed the presence of a lecithin:cholesterol acyltransferase (LCAT, EC 2.3.1.43) domain in these putative CsPDATs, suggesting that they belong to the LCAT superfamily (Pfam: 02450). Like all other PDAT proteins, several characteristic conserved regions were detected in the CsPDATs. For example, they contain a so-called lid domain that includes a disulphide bridge, which is possibly involved in destabilising the lipid bilayer, thus facilitating binding of the cleaved fatty acids to the active site of these enzymes (Martinelle et al., 1996; Peelman et al., 1998; Ståhl et al., 2004). A catalytic triad (Ser-Asp-His) conserved in all the PDATs (Ståhl et al., 2004) including the CsPDATs, is part of the catalytic domain of these enzymes, which transesterifies the fatty acid from PC to cholesterol to generate cholesterol ester. In all the PDAT proteins, a highly conserved domain III containing a salt bridge may be involved in phospholipid (PL) recognition, with several key residues within this domain responsible for its substrate specificity and binding (Peelman et al., 1998).

Collectively, these data obtained by comparison of CsPDATs with already characterised PDATs prove that the CsPDATs have distinct features typical of the PDAT family, and thus may function as PDATs to yield TAGs by transferring an acyl group from PLs to DAGs.

### Different *CsPDAT* members express in different camelina tissues

**Fig. 2.**
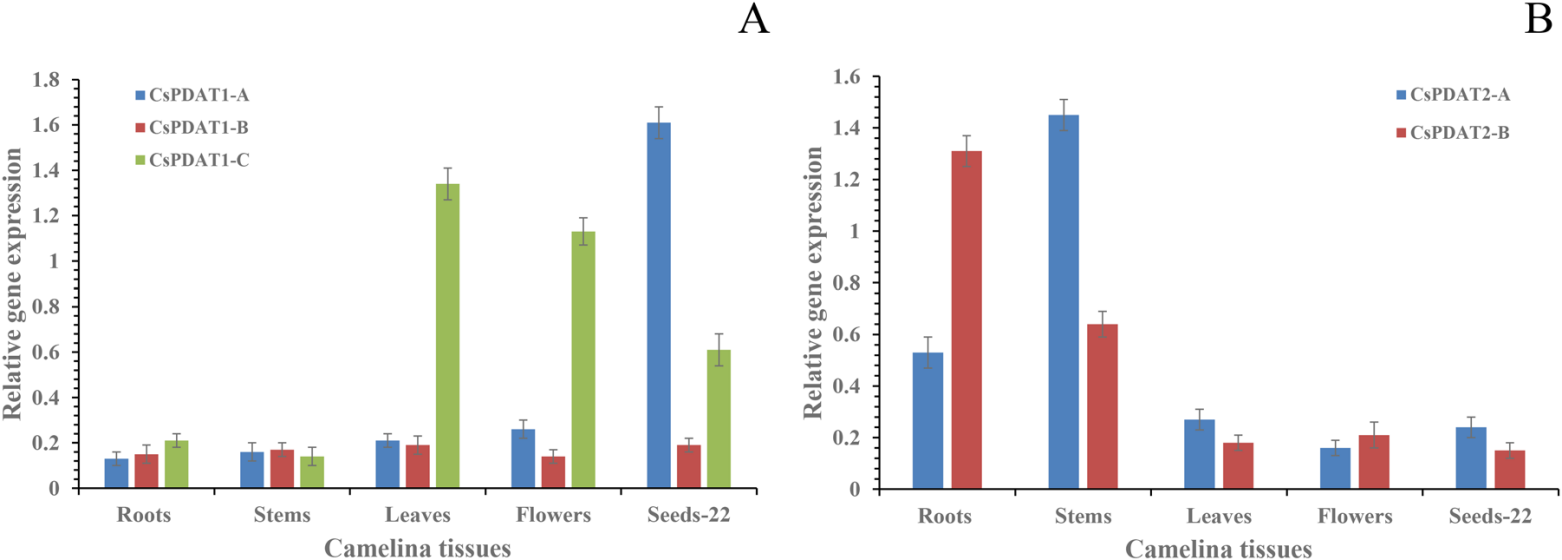
**qRT-PCR analysis of relative expression of camelina *CsPDAT* genes in different tissues.** (A) *CsPDAT1s* and (B) *CsPDAT2s* in seed, root, stem, leaf and flower tissues. Expressions were examined by qRT-PCR in different camelina tissues. Gene expression levels were normalised with respect to the internal control *β-actin* gene. Data bars represent the mean±s.e. level of relative transcript abundance of six replicates.

Expression profiling of genes encoding PDAT enzymes can help in identifying the PDAT's function in TAG biosynthesis in developing seeds and other tissues. To investigate the potential physiological roles of all candidate *CsPDAT* genes, real-time PCR was employed to examine their expression patterns in a range of organs including vegetative tissues, reproductive tissues, and middle-stage seeds [22 days after flowering (DAF)]. *CsPDAT1-A* transcript was detected preferentially in middle-stage seeds (22 DAF), whereas *CsPDAT1-C* mRNA was highly expressed in leaf and flower tissues, and to a lesser extent in seeds (22 DAF) (Fig. 2A). *CsPDAT2-A* expression was higher in stem than in root, and the opposite trend was observed for *CsPDAT2-B* (Fig. 2B). These data suggest that CsPDAT1-A is one of the major players in TAG biosynthesis in the seed, while CsPDAT1-C is mainly active in the leaf and flower. However, *CsPDAT2-A* and *CsPDAT2-B* were predominantly expressed in stem and root, respectively.

**Fig. 3.**
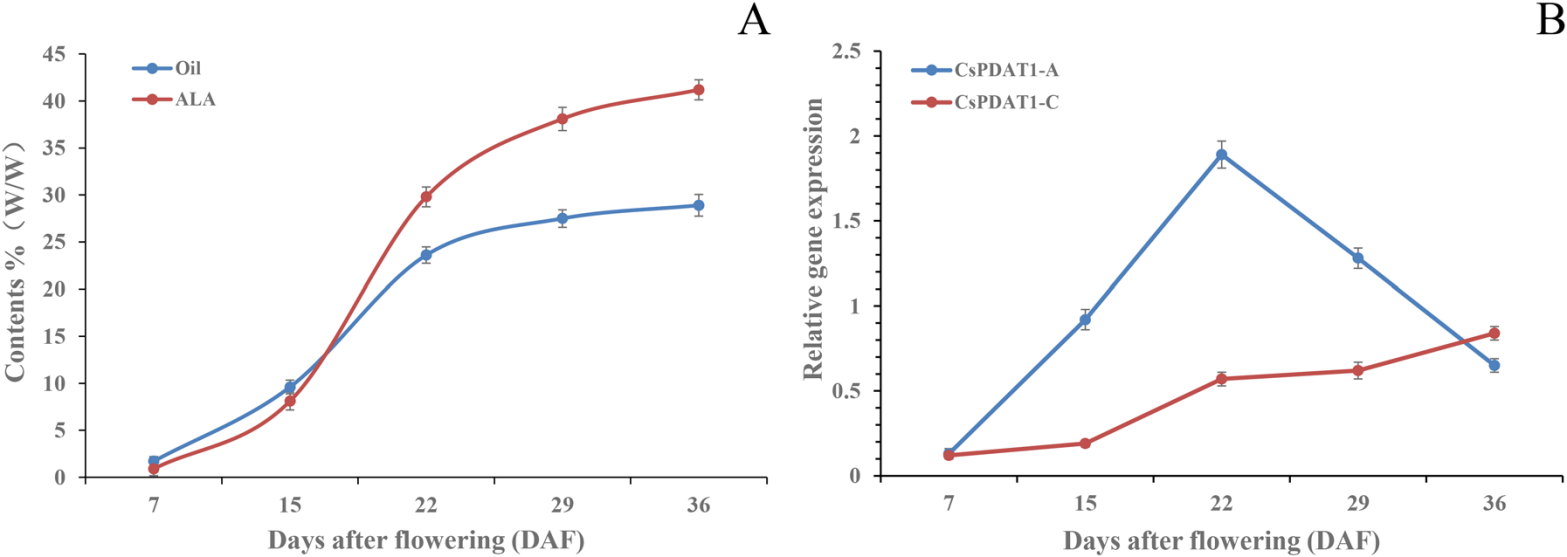
**Oil and ALA accumulation correlated with the expressions of *CsPDAT1-A* and *1-C* in camelina seed development.** (A) Total oil and ALA accumulation patterns during seed development (measured on 7, 15, 22, 29 and 36 DAF). The data are presented on a fresh weight basis (% of fresh weight) and shown as mean±s.e. (*n*=6). (B) Analysis of gene expression patterns of *CsPDAT1-A* and *CsPDAT1-C* during seed development revealed by qPT-PCR. Data are shown as mean±s.e. (*n*=6).

To further decipher the role of these *CsPDAT* genes in oil biosynthesis, we analysed the relationship between gene expression and seed oil and ɑ-linolenic acid (ALA; 18:3Δ9, 12, 15) accumulation patterns throughout seed development (Fig. 3A,B). During seed development, the oil content, on a fresh weight basis, fit a sigmoidal curve (*R*^2^=0.967), with the rapid phase of oil accumulation occurring between 15 and 29 DAF (Fig. 3A). A similar increase in accumulation pattern was detected for ALA content, on a fresh weight basis (Fig. 3A). The expression of *CsPDAT1-A* increased mostly in the early stages of seed development, with a peak at 22 DAF (Fig. 3B), simultaneous with the period in which oil and ALA accumulation occurred at the fastest rate (Fig. 3A). However, *CsPDAT1-C* mRNA displayed high expression in the later stages of seed development, with maximum levels achieved at 36 DAF (Fig. 3B), when the rate of oil and ALA accumulation had already reached a plateau (Fig. 3A). This result demonstrated that CsPDAT1-A, CsPDAT2-B and CsPDAT1-C might contribute differently to TAG biosynthesis during camelina seed development. Moreover, CsPDAT2-A, CsPDAT2-B and CsPDAT1-C may have unrelated physiological roles in camelina stem, root, and leaf and flower tissues, respectively.

### Different CsPDAT members function in different stress responses

In order to determine which member of the CsPDAT family is involved in camelina response to abiotic stresses, three-week-old seedlings grown under various stresses were harvested at a range of time points. The seedling samples were separated into two batches: one for total lipid extraction and TAG measurement and the other for total RNA extraction and quantitative real-time PCR (qRT-PCR) assay.

**Fig. 4.**
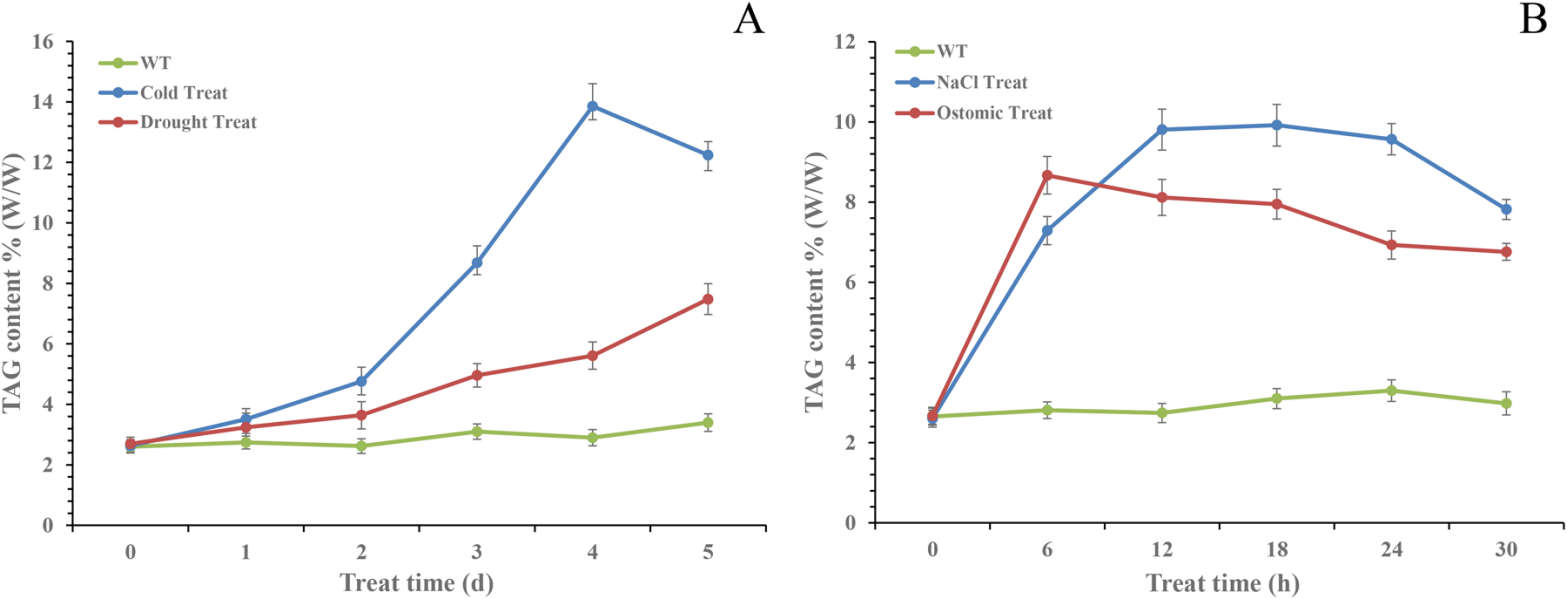
**TAG accumulation in camelina seedlings under various stresses.** The data are presented on a dried weight basis [% of dried weight (DW)] and shown as mean±s.e. (*n*=6). (A) TAG content of three-week-old camelina seedlings cultivated under cold and drought stresses for 0, 1, 2, 3, 4, and 5 days. (B) TAG content of three-week-old camelina seedlings cultivated under salt and osmotic stresses for 0, 6, 12, 18, 24, and 30 h.

TAG level in seedlings gradually increased following all stresses tested. However, peak levels and time points were different for different stressors (Fig. 4). The maximum amount (measured as folds of the control) of TAGs was 4.2-fold after 4 day (d) of cold (2°C) (Fig. 4A), 2.2-fold after 5 d of PEG6000-induced drought (Fig. 4A), 3.6-fold after 12 h of salt stress (200 mM NaCl) (Fig. 4B) and 3.1-fold after 6 h of osmotic stress (100 mM sorbitol) (Fig. 4B).

**Fig. 5.**
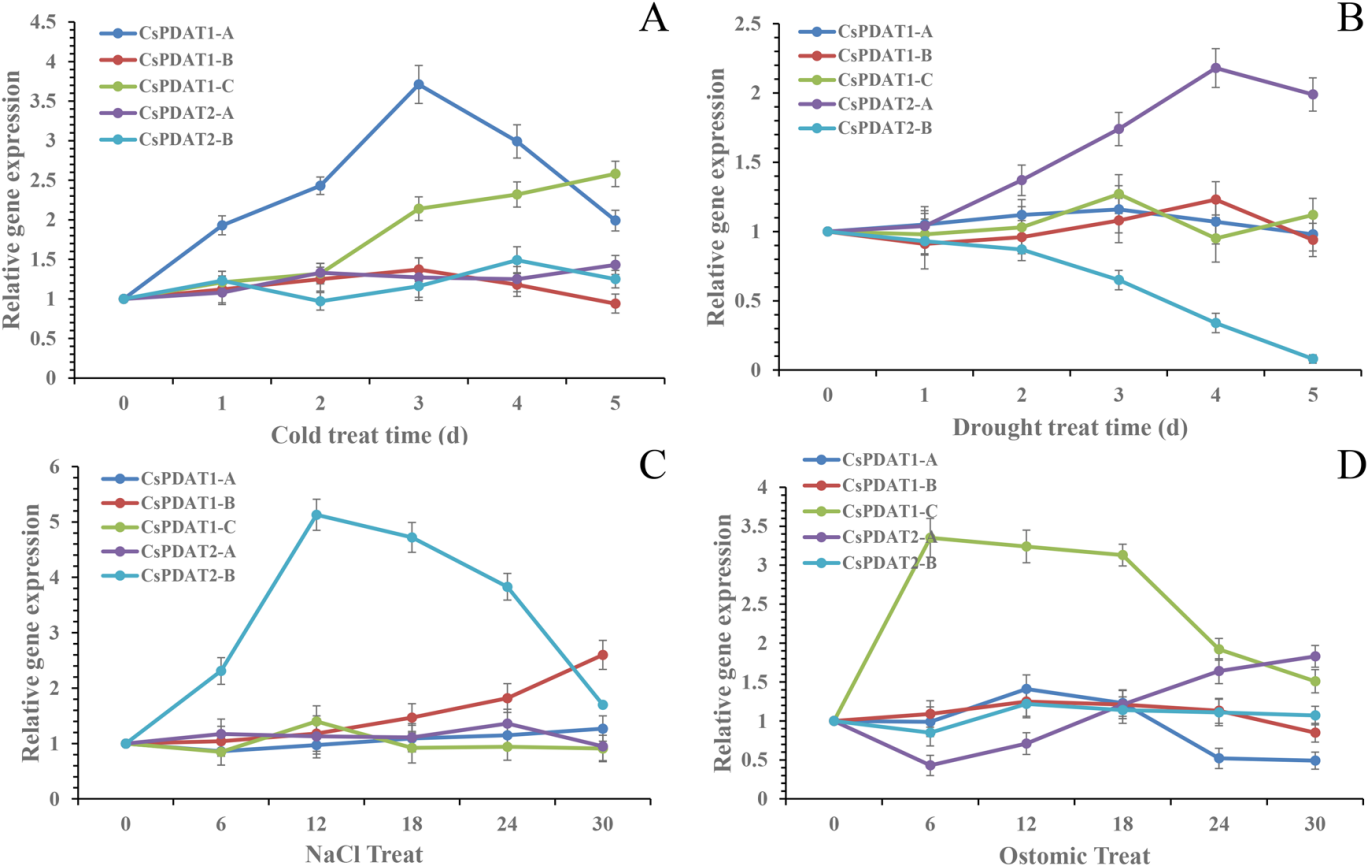
**Stress-responsive expression patterns of *CsPDAT* genes in three-week-old seedlings of camelina under abiotic stresses.** The relative mRNA levels were determined by qRT-PCR using *β-actin* as an internal control. Data represents the average of six independent experiments, and the error bars represent the standard deviations. Expression (fold) of *CsPDAT* genes in camelina subjected to cold stress (2°C) (A) or drought stress (15%PEG6000) (B) for 0, 1, 2, 3, 4, and 5 days, salt stress (300 mM NaCl) (C) or osmotic stress (100 mM sorbitol) (D) for 0, 6, 12, 18, 24, and 30 h.

If any member of the CsPDAT family functions in TAG accumulation in camelina seedlings under stress, the expression of this gene should vary correspondingly. To determine this variation, the gene expression of *CsPDATs* was examined using qRT-PCR. As shown in Fig. 5, CsPDAT members exhibited three different patterns of mRNA expressions in the seedlings during stress: up-regulation, down-regulation and no significant change from the control.

Following cold stress, *CsPDAT1-A* expression was up-regulated from 1 d until 4 d, with a peak level by 3 d, when it was approximately 3.5-fold higher than on 0 d (*P*<0.01). Another cold-upregulated gene was *CsPDAT1-C*, whose enhancement in expression began at 3 d, with the maximal level obtained at 5 d, which is higher than that of the control (0 d) by almost 2.6-fold (*P*<0.01) (Fig. 5A). Other *CsPDAT* genes showed no obvious changes during cold stress in comparison to the control (*P*<0.05).

Under drought stress (Fig. 5B), *CsPDAT2-A* expression increased from 1 d and reached the peak level on 4 d, with levels twofold higher than that in the control (0 d) (*P*<0.01). In contrast, *CsPDAT2-B* mRNAs reduced from 1 d to 4 d, with almost no transcript detected on 5 d (*P*<0.01). *CsPDAT1-C* expression showed slight, but not significant changes between control and stress conditions (*P*<0.05).

Salt stress greatly upregulated the expression of *CsPDAT2-B* by 5.1-fold at peak (12 h) over that of the control (0 h), with rapid increase observed between 6 and 12 h (*P*<0.01), and thereafter, gradually decreasing (Fig. 5C). *CsPDAT1-B* transcript showed slight increase under the stress, with the highest level detected at 30 h (*P*<0.01), lower than the peak level of *CsPDAT2-B* by 56%. A basal level of expression was observed for other *CsPDATs*, with no major change detected between the control and stress conditions (*P*<0.05).

Under osmotic stress (Fig. 5D), *CsPDAT1-C* mRNA was strongly induced to reach a peak level at 6 h, with amounts of transcript 3.2 times higher than at 0 h (*P*<0.05). In contrast, *CsPDAT*2-A showed reduction at 6 h, and then increased slowly through 24 to 30 h. The rest of *CsPDAT*s displayed basal expression during the stress.

Combined analysis of the expression patterns of *CsPDAT*s and TAG accumulation trends in camelina seedlings under four different types of stresses indicated that CsPDAT1-A, CsPDAT2-A, CsPDAT2-B and CsPDAT1-C may be the key contributors for TAG biosynthesis in response to cold, drought, salt and osmotic stresses, respectively.

### Transient expression of *CsPDAT* genes boosts accumulation of TAG in tobacco leaves

For further examination of each CsPDAT member's function in TAG biosynthesis, open reading frame (ORF) sequence of each *CsPDAT* gene was amplified from camelina and cloned into the pBI121 vector. The expression vector was then transformed into *Agrobacterium tumefaciens* strain GV3101. Finally, C*sPDAT* genes driven by CaMV 35S promoter in pBI121 vector were separately transformed for transient expression in tobacco leaves by *Agrobacterium* infiltration. The infected region and uninfected part of the tobacco leaf were sampled for total oil content and fatty acid composition on 7 d after Agro*-*transfection.

**Fig. 6.**
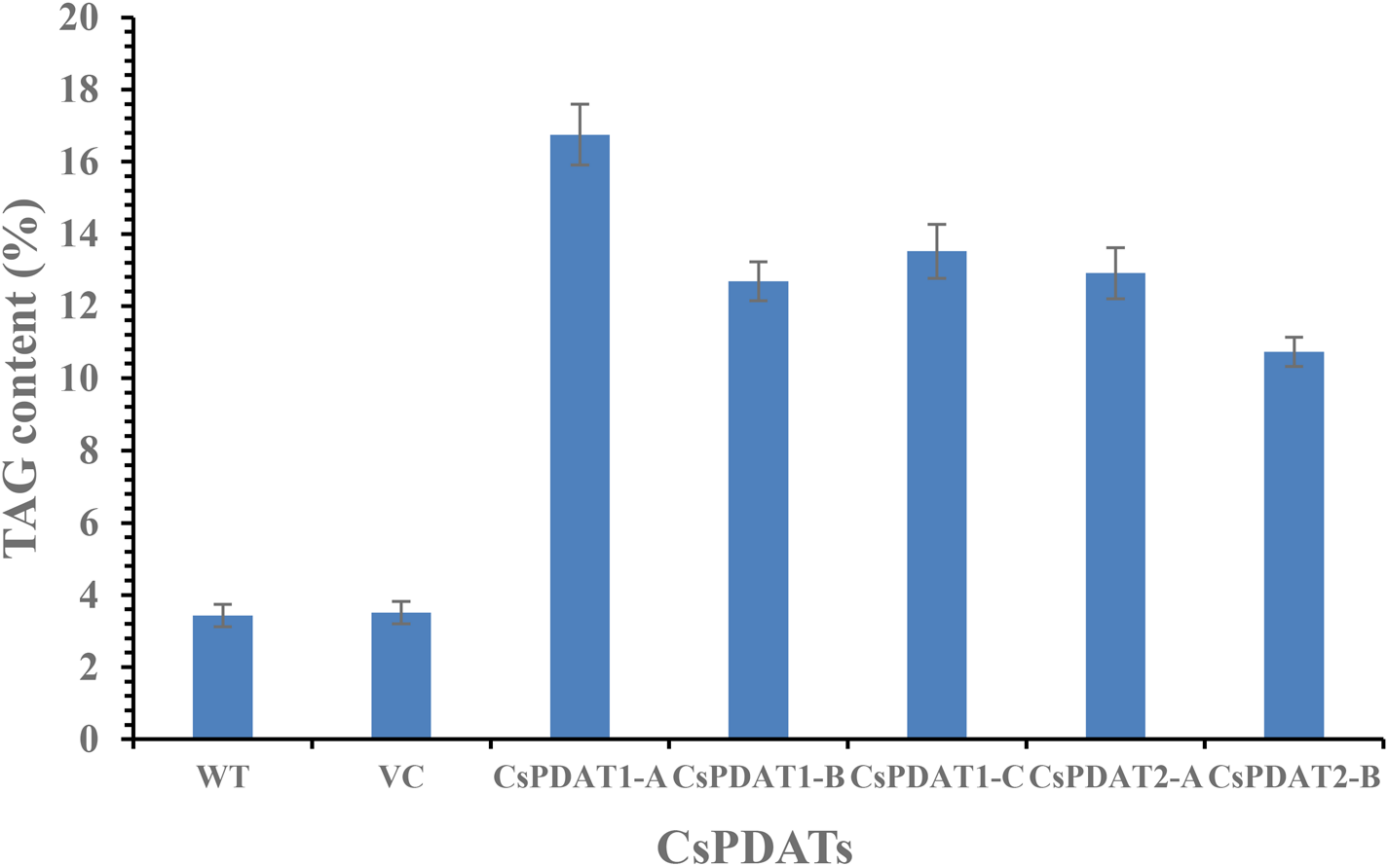
**TAG contents (*w/w*) in the tobacco leaf tissues where individual *CsPDAT* gene was transient expression separately.** Data represents the average of six independent replicates, and the error bars represents s.e. (*n*=6). WT, wild type; VC, empty vector control.

**Fig. 7.**
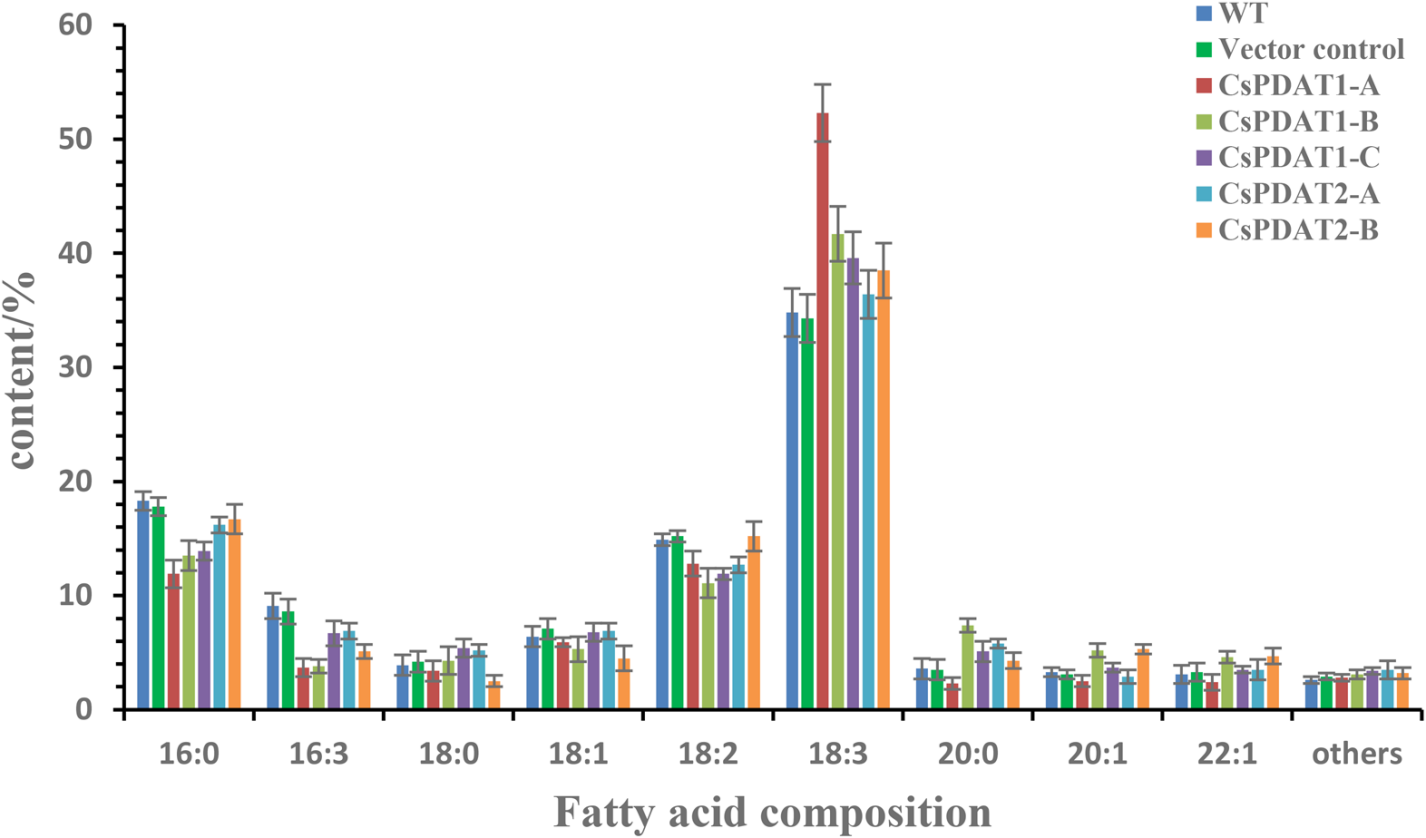
**The fatty acid profiles in the tobacco leaf tissue transient-expressing each *CsPDAT* gene.** 16:0, palmitic acid; 16:3, hiagonic acid; 18:0, stearic acid; 18:1, oleic acid; 18:2, linoleic acid; 18:3, linolenic acid; 20:0, arachidic acid; 20:1, eicosenoic acid; 22:1, erucic acid. Fatty acid composition in the leaf tissue was tested as described in materials and methods. Data represents the average of six independent replicates, and the error bars represents s.e. (*n*=6).

As shown in Fig. 6, transient expression of all *CsPDAT* genes led to significant enhancement in total oil content in the leaf, showing that TAG level was higher by at least threefold in the infected region than in the un-infected (control) and empty-vector infected parts. Moreover, *CsPDAT1-A* expression resulted in obvious changes in fatty acid composition in the leaf, with ALA showing an increase by 45%, and correspondingly lower levels of saturated fatty acids (16:0, 18:0 and 20:0) and linoleic acid (18:2), than in the controls (Fig. 7). However, significant variation in fatty acid profiles was not detected with transient expressions of the other *CsPDAT*s in the leaf tissue.

This transient assay in a heterologous system proved that each CsPDAT member identified here has enzymatic activity responsible for TAG accumulation *in vivo*, with CsPDAT1-A leading to selective accumulation of ALA-containing substrates.

## DISCUSSION

Camelina, a prominent oilseed crop, can accumulate high levels of ALA in its seed oil, serving as one of the most important sources of health-promoting vegetable oil rich in ALA and other plant-based oleochemicals. Understanding the metabolic pathway of oil synthesis is critical for genetic improvement of camelina oil quality and content, in order to increase its commercial production. The goal of this study was to identify members of the CsPDAT family in camelina genome, and to characterise their contribution to TAG biosynthesis in seed and non-seed tissues, particularly to investigate their functions in different physiological pathways, including abiotic stress response.

Information about plant PDATs is still very limited, although many studies have revealed the crucial role of PDATs in TAG biosynthesis (Wu et al., 2012; Zhang et al., 2009; Yuan et al., 2015). Taking advantage of the publicly available camelina genome sequence, we identified five *CsPDAT* members (Table 1 and Fig. 1) which could be grouped into two classes: *CsPDAT1* and *CsPDAT2*, namely *CsPDAT1-A*, *CsPDAT1-B*, *CsPDAT1-C*, *CsPDAT2-A*; and *CsPDAT2-B*, which supports the allohexaploid characteristic of camelina genome. Further bioinformatics analysis showed that these CsPDATs have several features conserved in all the plant PDATs, at both the gene and protein levels (Ståhl et al., 2004; Fan et al., 2013; Pan et al., 2015). For example, the exon/intron structures are conserved in most land plant *PDAT*s. The presence of a LCAT-like domain indicates that they belong to the LCAT superfamily. These PDATs are integral membrane proteins with a single TMD at the N-terminus. The C-terminal portion and the region between the TMD and the first LCAT-like motif are highly conserved. In addition, ER localisation signals were detected at the C-terminus suggesting that these CsPDATs may be localised in the ER, despite no subcellular localisation experiment conducted for CsPDATs. Moreover, it has been reported that some PDATs located on the plasma membrane (e.g. MiPDAT, and RcPDAT2) (Liu et al., 2016; Pan et al., 2015), while others are located in chloroplasts (e.g. CrPDAT) (Yoon et al., 2012). Therefore, further studies on the localisation of these CsPDAT protein paralogues are needed for their functional analysis.

In the present study, multiple members of both CsPDAT1 and CsPDAT2 subfamilies were detected, which is in agreement with the fact that multiple isoforms were also found for many other plant DGATs (Li et al., 2013; Pan et al., 2015), although the model plant Arabidopsis contains just one paralogue each for PDAT1 and PDAT2 (Ståhl et al., 2004). One question that naturally arises here is whether these multiple PDAT paralogues are evolved for highly redundant enzymatic activities, or do they lead to functional and expressional divergence?

To address this question, we first isolated cDNA clones encoding the five CsPDAT proteins from camelina, and subsequently, transient expression of each *CsPDAT* ORF driven by CaMV 35S promoter in PBI121 vector was achieved by *Agrobacterium* infiltration in tobacco leaves. Compared with the empty-vector and wild-type controls, the infected tobacco leaf regions accumulated much higher levels of TAGs (Fig. 6), suggesting that each of the five CsPDATs has the ability to synthesise TAG *in vivo*. These data indicate that the five *CsPDAT* genes are functional paralogues encoding active isoforms of PDAT enzyme in the camelina genome, which corroborates with previous reports where multiple functional paralogues were identified in flax (Pan et al., 2013) and castor bean (Kim et al., 2011).

Secondly, expression profiling of the five *CsPDAT* genes in various tissues of camelina plants (Figs 2 and 3) revealed that the transcripts accumulated in a tissue-specific pattern. *CsPDAT1-A* predominately expressed in middle-stage seeds with a peak at 22 DAF (Fig. 3B), which was positively correlated with rapid accumulation of oil and ALA in developing seeds (Fig. 3A). However, *CsPDAT1-C* mRNAs occurred abundantly in the later stages of seed development, with maximum levels observed at 36 DAF (Fig. 3B), the time period in which oil and ALA accumulation rate decreased (Fig. 3A). This result demonstrated that CsPDAT1-A and CsPDAT1-C might contribute differently to TAG biosynthesis during camelina seed development. Moreover, *CsPDAT1-C* was mainly expressed in leaf and flower tissues (Fig. 2A), whereas *CsPDAT2-A* and *CsPDAT2-B* were preferentially expressed in stem and root tissues, respectively (Fig. 2B), suggesting that CsPDAT1-C, CsPDAT2-A and CsPDAT2-B may have some other, yet unknown, physiological functions in addition to TAG synthesis in each of these non-seed tissues of camelina. Such tissue-specific expression was also reported for different PDAT members in flax (Pan et al., 2013), where *LuPDAT1/LuPDAT5* were highly expressed during the fast phase of lipid accumulation in the middle seed development stages, while *LuPDAT6* mRNA levels substantially increased during the initial stages of embryo development. However, *LuPDAT2* was found to be mostly expressed in flax vegetative tissues. In castor bean, *RcPDAT1-2* was exclusively expressed in developing seeds, whereas *RcPDAT1-1* was mostly expressed in all tissues with the exception of seeds (Kim et al., 2011). On the basis of the above mentioned findings, we propose that the existence of multiple paralogues of the *PDAT* gene family makes it conducive for the *PDAT*s to have expressional divergence, which appears to be a general trend in the evolution of the core eudicot PDATs (Li et al., 2005; Roth et al., 2007; Pan et al., 2015), although it is unclear how these multiple paralogues evolved in the plant genomes.

Thirdly, the present study demonstrated that the expression of different *CsPDAT* members was highly regulated by different stress treatments (Fig. 5), with a corresponding change in TAG content during the stresses (Fig. 4). This suggests that CsPDATs also play a role in camelina response to diverse environmental stresses. However, the induced expression patterns were different for different *PDAT* members under various kinds of stresses. Expressions of *CsPDAT1-A*, *CsPDAT2-A*, *CsPDAT2-B* and *CsPDAT1-C* were strongly upregulated by cold, drought, salt and osmotic stresses, respectively (Fig. 5). This again proves that the retention of multiple *PDAT* paralogues in plant genomes evolutionarily provides the basis for their functional and expressional divergence. Similarly, stress-induced TAG accumulation and gene expression was also detected for other TAG-synthesising genes, such as *DGAT* in Arabidopsis (Kong et al., 2013). Under stresses of ABA, jasmonic acid, salicylic acid, salt and sorbitol treatment, the expression of *AtDGAT1* is significantly induced in Arabidopsis seedlings, and TAG accumulation is increased too, indicating that TAG accumulation is an important stress response, and newly synthesised TAG, not incomplete storage oil degradation, accounts for TAG enhancement induced by various stresses. In the green alga *Chlamydomonas*, *DGAT1* and one of the *DGAT2s* show increased expression following nitrogen starvation, which induces TAG accumulation (Yoon et al., 2012). Contrastingly, in the diatom *Phaeodactylum tricornutum*, *PtDGAT1* was highly responsive to nitrogen starvation (Liu et al., 2016), whereas *PtDGAT2B* was strongly upregulated before the onset of TAG accumulation under nitrogen-replete conditions (Gong et al., 2013). Collectively, these findings confirm that induction of TAG-synthesis-related genes, leading to TAG accumulation, is important in stress response, despite the mechanism details being unknown.

Fourthly, fatty acid profiling (Fig. 7) showed that transient expression of *CsPDAT1-A* rather than the other *CsPDAT* members, resulted in a significant enhancement of ALA in the infected tobacco leaves compared to the control. This suggests that CsPDAT1-A may have high selectivity for ALA-containing substrates, which needs further experimental examination. Difference in substrate specificity has also been reported for other PDAT isoforms. For example, castor bean RcPDAT1A showed high specificity for ricinoleic acid, whereas RcPDAT1B and RcPDAT2 lacked this specificity (van Erp et al., 2011). Flax LuPDAT1 strongly preferred substrates containing ALA, but LuPDAT2 was highly selective for other polyunsaturated fatty acids (PUFAs) (Pan et al., 2013). Differential substrate preference described here once again supports functional divergence of multiple *PDAT* paralogues in higher plants.

In conclusion, our study provides the first comprehensive analysis on differential properties of the various CsPDAT family members in camelina, covering gene and protein structures, functional motifs, phylogenetic tree, cloning, spatial-temporal expression pattern, and transient expression in a heterologous system. Five members of the CsPDAT family were identified, sharing characteristic conserved features of plant DGAT proteins. The present data revealed that these CsPDAT isoforms varied in tissue-specificity and stress-induced expression as well as substrate selectivity, demonstrating that multiple PDAT paralogues offer the pathway for functional and expressional divergence of the PDAT family in plant genomes. This provides new insights into the mechanism underlying TAG biosynthesis and its regulation in camelina, thus benefiting future engineering projects aimed at enriching ALA or other PUFAs in plants and other organisms. Additional studies are necessary to further understand the differential physiological roles, besides TAG synthesis, of these CsPDAT members in various camelina tissues and in diverse stress responses.

## MATERIALS AND METHODS

### Camelina plant growth conditions and sampling

*C. sativa* cultivar SC-N1, commercially planted for 5 years in Taigu County, Shanxi province, China (E112.32°, N37.26°) was selected for this study. The plants were grown in a greenhouse at 23°C under natural light conditions supplemented with high-pressure sodium lights (250 µE m^−2^ s^−1^) with a 16 h light:8 h dark photoperiod. The plants were watered and fertilised under normal management. During the flowering stage, the emerging flowers were tagged, and flowers and seed pods were harvested at 7, 15, 22, 29 and 36 DAF to be used for qPCR and analyses of fresh/dry weight, fatty acid composition, and oil accumulation.

Fully developed leaves, stems and roots were collected from the 7-cauline leaf stage plants grown in half-strength Murashige and Skoog (MS) medium (PhytoTechnology Laboratories, Lenexa, Kansas, USA). For each experiment, tissue samples were harvested from at least six camelina plants. All collected samples were immediately chilled in liquid nitrogen and kept frozen at −80°C until their use in RNA extraction and other experimental measurements.

### Stress treatment and sampling

The same *C. sativa* cultivar was grown at 23°C under 16 h light:8 h dark cycle. The three-week-old soil-grown seedlings were selected for stress treatments. For cold stress treatment, the seedlings were placed at 4°C for up to 6 d under a 16-h photoperiod in a climatic chamber. Samples were collected at 0, 1, 2, 3, 4, and 5 d of the treatment. For drought stress, the seedlings were irrigated with 15% (w/v) PEG6000 solution. The culture conditions and sampling were done in the same manner as above. For salt and osmotic stress treatments, the seedlings were irrigated with solutions containing 150 mM NaCl and 100 mM sorbitol, respectively, in a climatic chamber. Samples were collected at 0, 6, 12, 18, 24, and 30 h after the stress.

All the samples were frozen immediately in liquid nitrogen, and then used for RNA extraction, oil measurement and other analysis. These experiments were repeated at least six times.

### Identification of PDAT genes from camelina genome and bioinformatics analysis

For genome-wide identification of PDAT genes, we conducted a TBLASTN search using the Arabidopsis AtPDAT1 and AtPDAT2 protein sequences identified previously (Ståhl et al., 2004), as queries against the camelina genomic database (www.ncbi.nlm.nih.gov/genome/?term=camelina+sativa). The genomic DNA, cDNA, and amino acid sequences corresponding to each putative PDAT were downloaded from the genome database.

Calculation of the theoretical molecular mass and pI values, primary structure analyses and topological organisation predictions were carried out by ExPASy proteomic tools (www.expasy.ch/tools/). The MEME program (http://meme.nbcr.net/meme/cgi-bin/meme.cgi) was employed to identify functional motifs in CsPDAT proteins using the default parameters, followed by Pfam analysis of the identified motifs for protein classification (Punta et al., 2012; http://pfam.sanger.ac.uk/search). The TMHMM (Krogh et al., 2001) program was used to predict TMD in candidate CsPDATs using the CBS Prediction Servers (www.cbs.dtu.dk/services/TMHMM-2.0/).

Phylogenetic analysis of amino acid sequences from camelina and other plant species was performed by the neighbour-joining method using the CLUSTALW multiple alignment program (Tamura et al., 2007). Molecular distances within the aligned sequences were calculated according to the position correction model. Branch points were tested for significance by bootstrapping with 1000 replications.

### Total RNA extraction, cDNA synthesis and qRT-PCR

Total RNA from each sample was isolated using the plant RNeasy mini kit (Sigma-Aldrich) according to the manufacturer's instructions. After extraction, RNA samples were treated with DNaseI (Promega) to remove contaminating DNA. RNA concentrations (ng/μl) and purity ratios (260/280 nm and 260/230 nm) were measured and calculated by NanoDrop 2000 spectrophotometer (Thermo Scientific).

Total RNA (5 μg) from each sample was used for cDNA synthesis using the First-Strand cDNA Synthesis Kit (Fermentas, Burlington, Ontario, Canada), with a cycling program of 42°C for 30 min in one cycle following manufacturer's procedure. The cDNA pools were quantified and then diluted to a final concentration of 100 ng/µl and used as templates for qRT-PCR.

All qRT-PCRs were carried out in an iCycler iQ™ detection system (Bio-Rad) using the intercalation dye SYBR Green I Master Mix kit (Applied Biosystems) as a fluorescent reporter. PCR controls were conducted in the absence of reverse transcriptase to ensure that RNA samples were free of DNA contamination. PCR reactions for each sample were performed in triplicates of each of the three independent biological replicates, in 25-μl volumes that included 1 μl of forward and reverse primer each (500 nM), 12.5 μl of SYBR green master mix, 5 μl of a 1:10 (v/v) dilution of cDNA, and 5.5 μl of HPLC molecular biology grade water. Reactions were performed in MicroAmp 96-well plates (Applied Biosystems) covered with optical adhesive covers (Applied Biosystems). The following program was applied: initial polymerase activation at 95°C for 10 min, and then a two temperature thermal cycle consisting of denaturation at 95°C for 15 s and annealing extension at 60°C for 1 min for a total of 30-40 cycles, and then a final extension at 72°C for 5 min.

Quantification of PCR products was performed by the 2-ΔΔCt calculation method, and the camelina β-actin gene was used as internal control to normalise the relative amount of mRNAs for all samples tested. The error bars represent the standard errors for the fold changes in relative gene expression calculated from three independent biological replicates and triplicate PCR reactions for each sample. The PCR primers for candidate genes quantified by qRT-PCR are listed in Table 2.

**Table 2.**
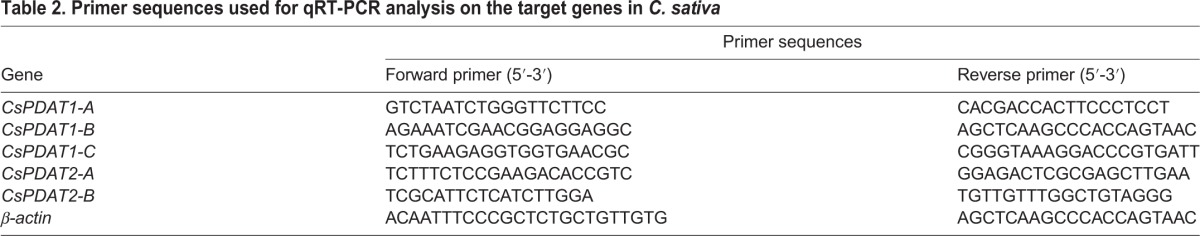
**Primer sequences used for qRT-PCR analysis on the target genes in *C. sativa***

### *CsPDAT* gene cloning, expression vector construction, and transient expression assay in tobacco leaves

Full-length cDNAs of each of the *CsPDAT1s* and *CsPDAT2s* were amplified by RT-PCR from total RNA of camelina developing tissues and then inserted into cloning vector (T-easy vector). After sequence verification, the vectors with correct sequence were used as templates to amplify each ORF sequence using Platinum *Taq* DNA Polymerase High Fidelity (Invitrogen). The primers representing sequences at the 5′ and 3′ termini of the ORFs from *CsPDAT* cDNAs are listed in Table 3. *Hin*dIII and *Bam*H1 restriction sites were added to each forward and reverse primer, respectively. The corresponding ORF of each *CsPDAT* was inserted into *Hin*dIII-*Bam*H1 digested pBI121 vector to generate the *35S*-driven expression vectors for each *CsPDAT* gene.

**Table 3.**
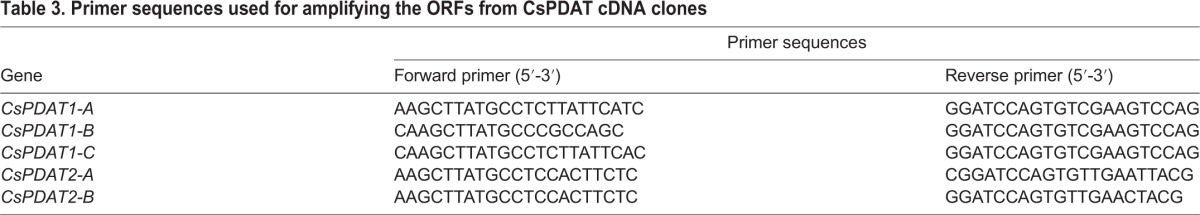
**Primer sequences used for amplifying the ORFs from CsPDAT cDNA clones**

The expression vector of each *CsPDAT* was then transferred into *Agrobacterium tumefaciens* strain GV3101. *Agrobacterium* cells were cultured overnight at 28°C. The cells were harvested and re-suspended using infiltration buffer (10 mM MES, pH 5.7, 10 mM MgCl_2_, and 150 mM acetosyringone), and subsequently, were separately used to infect abaxial side of leaves of four- to five-week-old *Nicotiana benthamiana* plants via vacuum infiltration for transient expression of *CsPDAT*s. At 7 d after infiltration, tobacco leaves (the infected regions and the corresponding un-infected regions) were sampled for lipid analysis.

### Lipid extraction and fatty acid analysis by gas chromatography

Lipids were extracted from camelina seeds and vegetative tissues using the method described by Xue et al. (2013). Briefly, 20-30 mg of seeds at different stages of development or vegetative tissues of camelina plants were freeze-dried for 48 h under high vacuum and weighed. The sample weight was recorded and the water content was determined as the weight difference of the samples before and after freeze-drying. The dried samples were placed in glass tubes, followed by addition of 1 ml freshly prepared sulphuric acid in methanol [5% (v/v)], 25 µl 0.2% BHT (butylated hydroxy toluene in methanol), 10 µg triheptadecanoin (17:0) (as an internal standard) and 300 µl toluene as co-solvent. The mixture was vortexed briefly, and then heated at 90-95°C for 1.5 h. After cooling down to room temperature, a mixture of 1.5 ml 0.9% NaCl (w/v) and 1 ml hexane was added for the transmethylation reaction. The homogenate was phase separated by centrifugation at 2000 rpm for 5 min. The upper organic phase containing fatty acid methyl esters (FAMEs) was transferred to a fresh tube, while the resulting aqueous phase was extracted with 2 ml hexane and the phases were separated again by centrifugation under the same conditions. The organic phases were combined, and dried using a flow of nitrogen gas. The nearly dried extracts were then dissolved in 1 ml hexane and 20 µl of the sample was transferred to gas chromatography (GC) vials.

The FAMEs were analysed on an Aglient7890B series GC system equipped with a HP-88 column (i.d. 0.25 mm×0.33 µm×10 m) and a flame-ionisation detector. The GC experiments were performed in triplicates (on three independent biological replicates). The fatty acids were identified by comparison of their retention times with those of the known standards. The oil content was quantified by comparing the concentrations of the fatty acids using the peak areas of the internal standard of known concentration. All data were analysed statistically. Significance of difference between pair-wise means was determined using a *t*-test.
